# Rival seminal fluid induces enhanced sperm motility in a polyandrous ant

**DOI:** 10.1186/s12862-018-1144-y

**Published:** 2018-03-23

**Authors:** Joanito Liberti, Boris Baer, Jacobus J. Boomsma

**Affiliations:** 1 Centre for Social Evolution, Department of Biology, University of Copenhagen, Universitetsparken 15, DK-2100 Copenhagen, Denmark; 2 Centre for Integrative Bee Research (CIBER), Department of Entomology, University of California Riverside, Riverside, CA 92521 USA

**Keywords:** Social insects, Sexual selection, Sperm competition, Sperm motility, Sexual conflicts

## Abstract

**Background:**

Promiscuous mating and sperm competition often induce arms races between the sexes with detrimental outcomes for females. However, ants with multiply-inseminated queens have only a single time-window for sperm competition and queens are predicted to gain control over the outcome of sperm storage quickly. The seminal fluid of *Acromyrmex* leaf-cutting ants reduces the viability of rival sperm, but how confrontations between unrelated ejaculates affect sperm storage remains unknown.

**Results:**

We investigated the effects of ejaculate admixture on sperm motility in *A. echinatior* and found that the proportion of motile spermatozoa, sperm swimming speed, and linearity of sperm movement increased when rival ejaculates were mixed in vitro. Major effects induced by the seminal fluid of rival males were of similar magnitude to those generated by queen reproductive tract secretions, whereas own seminal fluid induced lower sperm activation levels.

**Conclusions:**

Our results suggest that ant sperm respond via a self–non-self recognition mechanism to similar or shared molecules expressed in the reproductive secretions of both sexes. Lower sperm motility in the presence of own seminal fluid indicates that enhanced motility is costly and may trade-off with sperm viability during sperm storage, consistent with studies in vertebrates. Our results imply that ant spermatozoa have evolved to adjust their energetic expenditure during insemination depending on the perceived level of sperm competition.

**Electronic supplementary material:**

The online version of this article (10.1186/s12862-018-1144-y) contains supplementary material, which is available to authorized users.

## Background

While traveling through the reproductive tract of females after insemination, sperm typically experience environmental changes in temperature or pH, increasing or decreasing concentrations of organic molecules or emerging immune challenges [1]. These female factors often mediate viability selection of sperm and have led to the evolution of sophisticated olfactory recognition mechanisms that allow sperm to reach fertilization or storage sites despite of female-imposed handicaps [2, 3]. Sperm competition varies in intensity with degrees of female promiscuity [4], but has often selected for adaptations in sperm morphology [5, 6] and numbers of sperm produced [7], implying that male testis size relative to body size [8–11] and higher rates of spermatogenesis [12] are common markers of sperm competition. However, paternal success in sperm competition is not only dependent on quantitative measures of sperm numbers but also on qualitative parameters such as optimal motility to reach the eggs or sperm-storage organs first while minimizing undue viability costs [13–16].

Because seminal fluid is ejaculated together with sperm, these glandular secretions can play a key role in securing sperm viability and paternity in female tracts where interactions between secretions by females and rival males set the rules for sperm competition. In particular, seminal fluid is expected to enhance the success of own sperm (sperm capacitation) and to reduce the success of alien sperm (sperm incapacitation) [17, 18]. Known effects of seminal fluid include prolonging the survival of own sperm [19, 20] and the selective elimination of rival sperm [21], but effects on sperm motility are also increasingly identified, both for internally and externally fertilizing species. For example, male fowls (*Gallus gallus*) can adjust the velocity of their spermatozoa through allocating more seminal fluid when mating with more attractive females [22] and male morphs of externally fertilizing grass gobies (*Zosterisessor ophiocephalus*) and Arctic charr (*Salvelinus alpinus*) produce seminal fluids that differentially enhance sperm motility [23–25]. In Arctic charr it was also shown that own seminal fluid has an inhibitory effect on sperm motility activation, relative to rival male seminal fluid or water controls [26], implying that sperm motility is regulated by a self–non-self recognition mechanism. Taken together, these studies highlight that vertebrate sperm have been selected to adjust their motility parameters based on compositional differences in male and female reproductive fluids that reflect levels of sperm competition, but to our knowledge such forms of sperm sensing and regulation of sperm motility have never been documented in invertebrates.

The males of evolutionarily derived ants have extremely short life spans and die shortly after their mating flight during which they copulate with one or more females (queens). They secure their reproductive success via stored sperm in the spermatheca of queens, who can live for decades in some species and produce thousands to millions of offspring [27, 28]. Once inseminated, ant queens never re-mate later in life, so the number and quality of sperm initially stored set an upper limit to their lifetime reproductive success [29]. Stored sperm therefore needs to retain viability for a similar time span, imposing strong selection on males to produce ejaculates of high quality and on queens to continue sperm preservation after storage [21, 29]. This mutual effort is completely devoid of sexual conflict under strict lifetime monogamy, the ancestral situation in ants [27], but becomes contentious in lineages where queens secondarily evolved obligate polyandry, because being inseminated by a series of males during the same mating flight inevitably results in sperm competition [28]. Remarkable adaptations produced by these selective pressures include the aggregation of sperm in cooperative bundles to enhance sperm swimming velocity in the desert ant *Cataglyphis savignyi* [30], the capacity of male seminal fluid to incapacitate sperm from rival males in both *Atta* and *Acromyrmex* leaf-cutting ants [21], and the evolution of queen reproductive tract fluid that enhances sperm motility, and thus storage of viable spermatozoa, in *Acromyrmex echinatior* [31].

The *Atta* and *Acromyrmex* leaf-cutting ants evolved ca. 15 million years ago from ancestors with exclusively singly-mated queens [32], but they elaborated their polyandrous life-histories in very different directions. *Atta* queens have a massively enlarged spermatheca relative to the pre-storage organ, the bursa copulatrix, and ejaculates become almost immediately deposited in this specialized organ [33]. The virgin queen sexual tract, including the small bursa and huge spermatheca, has hardly any fluid (J. Liberti, unpublished observations) so *Atta* sperm are unlikely to actively move in secretions other than own seminal fluid before reaching their final storage destination. In contrast, *Acromyrmex* queens have retained the ancestral reproductive biology of attine fungus-growing ants, where males transfer ejaculates to an enlarged and fluid-filled bursa copulatrix, after which each sperm needs to swim to reach the spermathecal duct in competition with sperm from other ejaculates [34]. Ultimate female (queen) control of sperm competition reflects this difference. Spermathecal secretions terminate mutual sperm incapacitation induced by seminal fluid very shortly after insemination in *Atta* [21], but seminal fluid is unlikely to ever enter the spermatheca in *Acromyrmex* so queens are not expected to have evolved such mechanisms in this genus. Consistent with the necessity to move individually, we recently showed that reproductive tract secretions of *Acromyrmex* queens enhance sperm motility in vitro, which likely reflects the existence of a chemokinetic gradient facilitating storage of the most viable sperm [31]. However, the timing and location of this process and the possible interactions with own and non-own seminal fluids have remained unclear.

In the present study, we resolve some of these questions using a series of experiments to quantify how seminal fluid affects sperm motility in *Acromyrmex* leaf-cutting ants. We first assessed the overall effect of sperm competition on sperm motility by mixing ejaculates of different males in vitro and found that motility was substantially enhanced after exposure to seminal fluid from multiple males. We then quantified the effects of a rival male’s seminal fluid on sperm motility while differentiating between sperm with and without own seminal fluid, and we compared the magnitude of these responses with the known sperm-motility enhancing effect [31] induced by fluid from the queen reproductive tract. We infer that these conditional increases in sperm motility are likely to be costly adaptations; this response is instrumental for success in sperm competition in the bursa copulatrix where sperm are provisionally stored, but may also induce reactive oxygen species (ROS) damage. Such a trade-off could then negatively affect sperm viability after final storage in the spermatheca where reduced sperm viability compromises the lifetime reproductive success of queens.

## Methods

Colonies of *Acromyrmex echinatior* leaf-cutting ants were collected in Gamboa, Panama, between 2002 and 2014 (Additional file 1: Table S1) after obtaining collection and export permissions from the Autoridad Nacional del Ambiente y el Mar (ANAM), and were then reared under controlled laboratory conditions of 25 °C and RH 60–70% at the University of Copenhagen. In all experiments we used a microscopy system and analysis pipeline that enabled us to simultaneously measure a set of *A. echinatior* sperm motility parameters [31]. Spermatozoa were stained in a solution of Hayes saline (9 g NaCl, 0.2 g CaCl_2_, 0.2 g KCl and 0.1 g NaHCO_3_ in 1000 ml H_2_O, adjusted to pH 8.7 and sterilized by filtration through a 0.22 μm syringe-filter, Membrane Solutions), containing a cell-permeant nucleic acid stain (SYTO 13, Molecular Probes) at a concentration of 375 μM, which pilot experiments and our previous study [31] established to be the minimum concentration required for clearly identifying sperm heads with our microscopy system. These mixtures were pipetted into a counting chamber (SC-20-01-04-B, Leja) and observed two minutes later with a spinning-disk confocal microscope (Revolution XD, Andor). To do this we used a 20× dry objective and excited the dye with a 488 nm laser, recording motility for 5 s at 30 frames per second (fps) with an Andor iXon DU-897-BV EMCCD camera. For each experiment we performed ten trials while randomizing treatment loadings on slides, and every experiment was performed twice with the same colony combinations (Additional file 1: Tables S2-S5). In each trial, we performed two series of recordings by moving the field of view sequentially across slide chambers. Video recordings were analyzed with the computer assisted sperm analyzer (CASA) plugin [35] for ImageJ (http://imagej.nih.gov/ij/) using the same parameter settings that we previously determined [31], which are specific to *A. echinatior* and the microscope system used: a = 20, b = 250, c = 30 d = 12, e = 3, *f* = 10, g = 10, h = 5, i = 1, j = 10, k = 10, l = 10, m = 80, *n* = 80, o = 50, *p* = 60, q = 30, r = 683.3728, s = 0, t = 1, u = 1.

We obtained the following sperm motility parameters: Proportion of motile sperm: the proportion of tracked sperm identified as exhibiting motility during the 5 s of analysis; Curvilinear velocity (VCL): point to point distance traveled by sperm over the 5 s of analysis, averaged to a per second value; Velocity average path (VAP): velocity over an average path generated by a roaming average of sperm position from one-sixth of the video’s frame rate (30 fps), where each point is obtained by averaging the coordinates of a set number of locations on the VCL path; Velocity straight-line (VSL): velocity measured using the first point on the average path and the point reached that is furthest from this origin during the 5 s of observation. Linearity (LIN): the VSL/VAP ratio, describing path curvature. The CASA plugin only provides velocity and linearity values for motile spermatozoa. For videos where all sperm cells were non-motile, we considered velocity and linearity values to be zero because the same ejaculates were motile in glandular secretion treatments on the same slides, so that lack of motility represented biologically relevant results [31].

### The effect of ejaculate admixture on sperm motility parameters

**Fig. 1:**
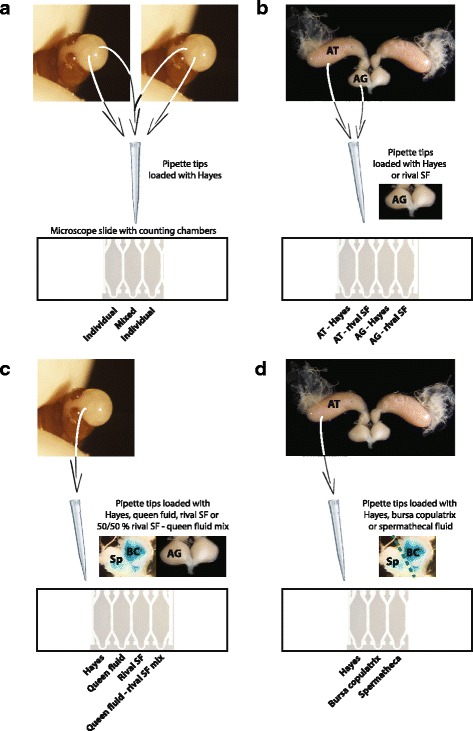
Design of the experiments testing the effects of (**a**) ejaculate admixture, (**b**) own and rival male seminal fluid, (**c**) Hayes, queen reproductive tract fluid, rival seminal fluid and a 50/50% mixture of these fluids, and (**d**) Hayes, bursa copulatrix and spermathecal fluid on *A. echinatior* sperm motility parameters. AT = accessory testes; AG = accessory glands; Sp = spermatheca; BC = bursa copulatrix; SF = seminal fluid

Sperm motility parameters were recorded from paired males collected from different (unrelated) colonies, both individually (i.e. exposed only to own seminal fluid) and combined (i.e. exposed to both own and alien seminal fluid; fig. 1a). Ejaculates were obtained by separating the male gasters from their mesosomas and gently increasing pressure from the anterior to the posterior side of the gaster using thumb and forefinger, in a similar way as has been used for larger males of *Atta* leaf-cutting ants [36, 37]. Single 20 μl pipette tips previously loaded with 3 μl Hayes saline containing SYTO 13 (375 μM concentration) were briefly dipped either twice in one of the two individual male ejaculates or sequentially in the two different ejaculates. The three sperm-containing fluids (two individual and one mixed treatments) were immediately pipetted into three counting chambers of the same four-chamber slide (SC-20-01-04-B, Leja) allowing sperm motility to be recorded two minutes after loading the slides and within four minutes from ejaculation. In doing so, we were able to equalize the amount of sperm pipetted across treatment groups as the number of tracked sperm in the subsequent analyses did not differ between the individual and mixed groups (*F*_1,118_ = 2.05, *P* = 0.15).

### The effects of own and alien seminal fluid on sperm motility

To assess the effect of own and rival seminal fluid on sperm motility we first obtained seminal fluid from a single male by pulling the last abdominal sclerites with watchmaker forceps until the accessory glands (AGs) were exposed, after which we separated these from the accessory testes (ATs) and placed them into 10 μl Hayes in a 0.2 ml PCR tube [21, 38]. The two AGs were punctured with watchmaker forceps and vortexed vigorously for 30 s so that any suspended sperm would be pelleted in the subsequent centrifugation step at 17,000 g for 3 min at room temperature. We then transferred 6 μl supernatant to a new 0.2 ml PCR tube, vortexed and centrifuged as before, after which 3 μl supernatant was placed into 4 μl Hayes containing SYTO 13 (final concentration 375 μM) and two aliquots of 3 μl of this solution were used as “rival seminal fluid” test fluids (see below). Two 3 μl aliquots of control solution with only Hayes saline were created in parallel using the same centrifugation procedures. We obtained these fluids freshly for each trial and always used them within 20 min after collection.

Immediately after preparing these solutions, we dissected a male from a different colony (Additional file 1: Table S3) in a droplet of Hayes saline until exposing the reproductive tract (fig. 1b). Spermatozoa were collected by puncturing either the ATs (containing sperm deprived of own seminal secretions) or the AGs (containing sperm suspended in own seminal secretions, as the AGs are connected to the ATs in leaf-cutting ants, and become filled with sperm prior to ejaculation and prior to dissection [21, 36]), and briefly dipping 20 μl pipette tips loaded with 3 μl of the previously prepared rival seminal fluid solutions or the Hayes-only control solution (see previous paragraph) in the outflowing sperm. For each focal male, sperm suspensions were immediately pipetted in the same four-chamber slide (SC-20-01-04-B, Leja) to produce four parallel treatment combinations: (i) sperm collected from one AT swimming in Hayes saline, (ii) sperm collected from the other AT swimming in Hayes-diluted rival seminal fluid, (iii) sperm collected from one AG swimming in Hayes saline, and (iv) sperm collected from the other AG swimming in Hayes-diluted rival seminal fluid (fig. 1b). Sperm motility parameters were subsequently recorded two minutes after loading the slides as explained above. Dissections of focal males never took more than five minutes so that we always recorded sperm motility within ca. seven minutes from dissection.

### Comparing the effects of seminal fluid and queen reproductive tract fluid on sperm motility

To compare the effects of rival seminal fluid and queen reproductive tract fluid we first collected these secretions as described above and in the literature [21, 31]. We took an equal volume of each and mixed these fluids in an additional tube to produce a 50/50% treatment. Finally we produced a Hayes saline control and tested the ejaculated sperm of the same male against these four treatments (fig. 1c). To obtain queen reproductive tract fluid, a virgin queen from a colony unrelated to the colony from which the focal male was sampled (Additional file 1: Table S4), was dissected under a stereo microscope in a droplet of Hayes saline. The bursa copulatrix and the attached spermatheca were separated from the rest of the reproductive tract, cleaned to remove any fat body tissue, and placed together in 5 μl Hayes in a 0.2 ml PCR tube. The tube was centrifuged for 3 min at 17,000 g at room temperature and 3 μl supernatant was transferred into a new tube, after which 1.5 μl was added to 2 μl Hayes containing SYTO 13 (375 μM final concentration) in a 0.2 ml tube, to produce a queen-reproductive-tract-fluid-only treatment.

Another 0.75 μl of the same supernatant was added to a separate 0.2 ml tube containing 2 μl Hayes with SYTO 13, to which we added an equal volume of rival male seminal fluid. AG secretions were obtained as described above and 1.5 μl of this fluid was added to 2 μl Hayes containing SYTO 13 to produce a rival-male-seminal-fluid-only treatment, while 0.75 μl was added to the previously prepared tube containing the same amount of queen reproductive tract fluid, thus producing a 50/50% mix of queen fluid and seminal fluid. A fourth control treatment was prepared with only Hayes containing SYTO 13 at the same 375 μM concentration. We obtained also these fluids freshly for each trial and used them within 20 min from dissections. Single 20 μl pipette tips were loaded with 3 μl of each of these four fluids and were sequentially dipped into the same male ejaculate, after which the sperm-containing fluids were randomly pipetted into the four chambers of a single microscope slide (SC-20-01-04-B, Leja). Sperm motility was then recorded as explained above, two minutes after loading the slides and within four minutes from ejaculation.

To establish the source of the active compounds in the female reproductive tract, virgin queens were dissected as described above, but this time their reproductive tracts were further separated into spermatheca and bursa copulatrix (fig. 1d), which were each placed into 3 μl Hayes in separate 0.2 ml PCR tubes (see Additional file 1: Table S5 for colony sampling combinations). The tubes were centrifuged for 3 min at 17,000 g at room temperature and 1.5 μl supernatants were transferred into new tubes containing 2 μl Hayes with SYTO 13 (375 μM final concentration). A control with only Hayes saline and SYTO 13 at the same concentration was produced in parallel, after which 3 μl of these different fluids were loaded in separate 20 μl pipette tips. These tips were sequentially dipped in outflowing sperm after puncturing the same male ATs, and these sperm-containing fluids were randomly loaded in three counting chambers within the same slide (SC-20-01-04-B, Leja). Also here, queen fluids were freshly obtained for each trial and used within 20 min from queen dissection. Sperm motility was recorded once more as previously described, two minutes from loading the slides and within ca. seven minutes from dissection of focal males.

### Statistical analyses

As CASA yields sperm velocity measures with substantial intercorrelations [31, 39], a Principal Component Analysis (PCA) was performed in JMP v. 12, incorporating curvilinear velocity (VCL), velocity on the average path (VAP) and straight-line velocity (VSL). The first principal component (PC1) of these three motility measures was subsequently used as a proxy for overall sperm velocity in the subsequent statistical analyses. Apart from PC1, we also analysed the proportion of motile sperm and linearity of sperm motility (LIN; the ratio between VSL and VAP, capturing path curvature) as dependent variables in linear mixed-effects models fitted by restricted maximum likelihood. Each of the four experiments described in the previous sections consisted of ten trials, which were replicated once with identical colony combinations, so that trial and experimental replicate were treated as random effects, while treatment, time point (the two consecutive series of video recordings for each trial) and their interaction term were treated as fixed effects. The datasets used in statistical analyses can be found in Additional file 2.

## Results

**Fig. 2:**
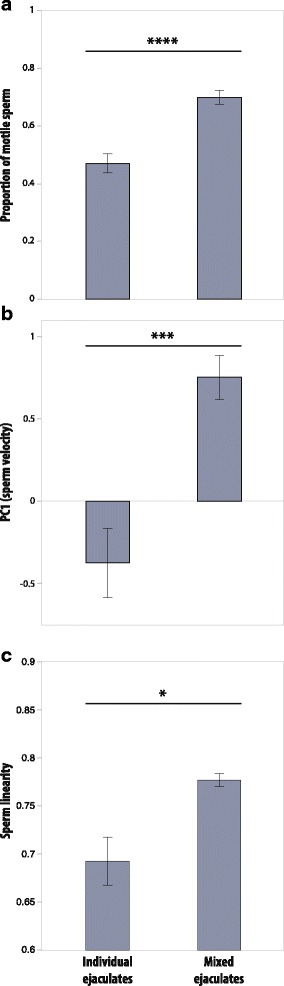
When ejaculates of two distinct *A. echinatior* males are mixed in vitro, (**a**) a higher proportion of spermatozoa are actively motile (**** *P* < 0.0001), (**b**) sperm swim faster (*** *P* < 0.001), and (**c**) sperm move more linearly (* *P* < 0.05) than when motility is assessed within the same ejaculates without contact with non-own seminal fluid

Mixing ejaculates (sperm and seminal fluid) from two males increased the number of motile sperm by 50% compared to non mixed samples of each male (fig. 2a; *F*_1,106_ = 22.49, *P* < 0.0001). This increase in motile sperm was correlated with a > 20% average increase in composite sperm velocity as captured by PC1 (fig. 2b; *F*_1,106_ = 13.69, *P* = 0.0003), similar to the separate variables that loaded PC1 (curvilinear velocity VCL = 21.6%; velocity on the average path VAP = 22.6%; straight-line velocity VSL = 25.5%), and an increase in sperm linearity (LIN; fig. 2c) of 11.6% (*F*_1,106_ = 5.54, *P* = 0.0204; see Additional file 1: Table S6 for details).

**Fig. 3:**
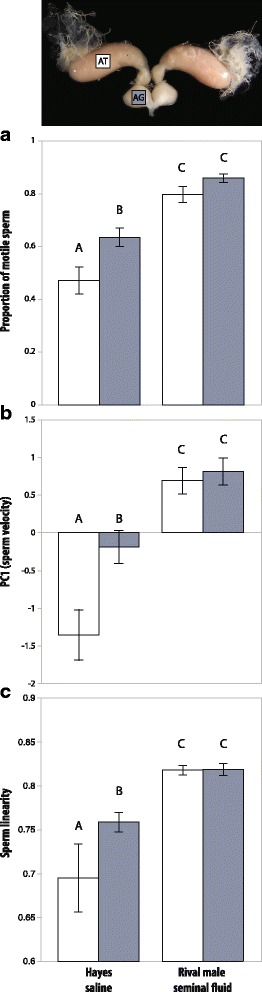
Effects rival male seminal fluid on sperm motility parameters in *A. echinatior* with (blue bars) and without (white bars) own seminal fluid being present, which depended on whether we dissected accessory testes (AT) or accessory gland (AG) material (top picture). (**a**) Own seminal fluid had a positive effect on the proportion of motile sperm as compared to sperm deprived of any seminal fluid (first two bars; *F*_1,142_ = 8.69, *P* = 0.0037), but the highest proportion of motile sperm was found in the presence of rival male seminal fluid, irrespectively of own seminal fluid being present or not (last two bars compared to first two bars; *F*_1,142_ = 79.78, *P* < 0.0001). (**b**) Sperm swimming in Hayes saline were faster when collected from the accessory glands (with own seminal fluid) than from the accessory testes (first two bars; *F*_1,142_ = 13.81, *P* = 0.0003), and the highest velocity was found when seminal fluid from a rival male was present (last two bars compared to first two bars; *F*_1,142_ = 48.03, *P* < 0.0001). (**c**) Sperm linearity was greatest when seminal fluid from a rival male was present (last two bars compared to first two bars; *F*_1,142_ = 21.00, *P* < 0.0001) and own seminal fluid induced more linear sperm movement than controls without any seminal fluid (first two bars; *F*_1,142_ = 5.00, *P* = 0.0269). All bars represent means ± SE and levels not connected by the same letter are significantly different (Student’s *t* tests)

Exposure of sperm to own seminal fluid significantly increased the proportion of motile sperm by 29.8%, significantly increased sperm swimming speed by 20% (VCL = 24.2%; VAP = 22.2%; VSL = 21.2%), and made sperm swim 10.1% more linearly compared to sperm in the control treatment not containing any seminal fluid (fig. 3, first two bars in panels a, b and c, and Additional file 1: Table S7). However, motility values were much higher in samples where sperm were exposed to seminal fluid of rival males independently of own seminal fluid being present or not. We observed an additional increase of ca. 40% in the proportion of motile sperm, a ca. 15% further increase in swimming speed, and a ca. 8% further increase in linearity compared to when only own seminal fluid was present (fig. 3 and Additional file 1: Table S7).

**Fig. 4:**
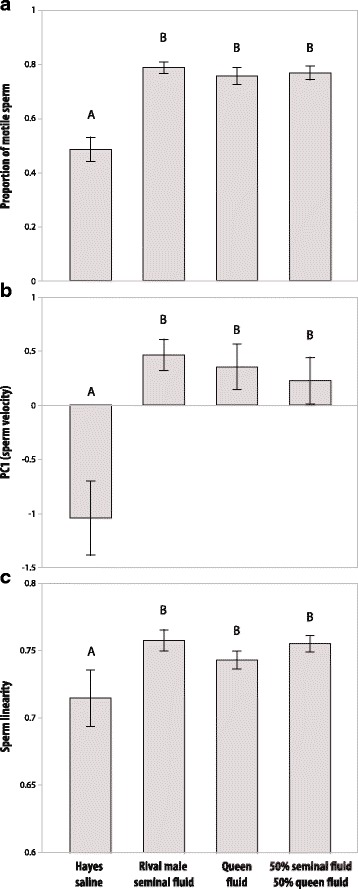
Comparison between the effects of own seminal fluid, a rival male’s seminal fluid, a virgin queen’s reproductive tract fluid, and a mixture of equal volumes of the same rival male’s seminal fluid and queen’s reproductive tract fluid on sperm motility parameters in natural ejaculates of *A. echinatior* males. (**a**) A higher proportion of spermatozoa were active when in contact either with rival male seminal fluid, queen reproductive tract fluid or a 50/50% mixture of rival male seminal fluid and queen reproductive fluid, as compared to sperm only exposed to own seminal fluid (*F*_1,142_ = 71.98, *P* < 0.0001), but all these treatments were equally effective in activating spermatozoa. These similar increases in sperm motility were also reflected by increases in (**b**) sperm velocity (*F*_1,142_ = 31.54, *P* < 0.0001) and (**c**) sperm linearity (*F*_1,142_ = 7.79, *P* = 0.0060). Bars are means ± SE and levels not connected by the same letter were significantly different in post-hoc Student’s *t* tests

We previously showed that secretions of *A. echinatior* queen reproductive tracts increase sperm motility [31], similar to what we now found for seminal fluid. To test whether the two secretions have additive effects we next quantified the effects on sperm motility of seminal fluid and female secretions separately or in combination. We confirmed that rival male seminal fluid and queen reproductive tract fluid both increase sperm motility and without any significant difference between the two secretions. Furthermore, we found no further increases in sperm motility in the sperm samples exposed to both secretions at the same time (fig. 4 and Additional file 1: Table S8).

**Fig. 5:**
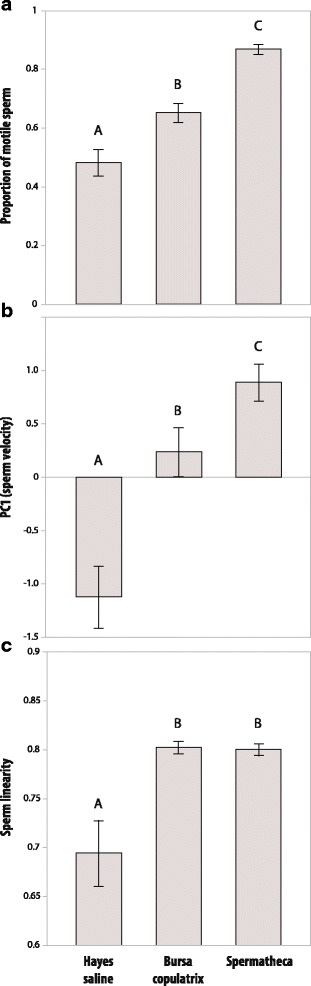
Effects of the different compartments of *A. echinatior* queen reproductive tract on sperm motility. (**a**) Fluid sampled from the final sperm storage organ of queens (spermatheca) activated a higher proportion of spermatozoa than the bursa copulatrix pre-storage organ (*F*_1,105_ = 25.90, *P* < 0.0001) and these effects were proportional to (**b**) higher sperm velocity in spermathecal fluid as expressed by PC1 (*F*_1,105_ = 5.71, *P* = 0.0187). However, linearity in sperm motility (**c**) was equally enhanced in spermathecal and bursa copulatrix fluids (*F*_1,105_ = 0.01, *P* = 0.93). Bars show mean ± SE and levels not connected by the same letter were significantly different in post-hoc Student’s *t* tests

Finally, we assessed the respective effects of fluids sampled from the spermatheca and bursa copulatrix on sperm motility and found that only the spermathecal fluid induced the maximal sperm motility increase comparable to the increase mediated by alien seminal fluid. The bursa copulatrix fluid induced a weaker motility enhancement albeit still significantly higher than the Hayes saline controls. However, sperm linearity was enhanced in equal measure by both fluids, suggesting this induction is qualitative while the proportion of motile sperm and sperm velocity responded to a quantitative factor (fig. 5 and Additional file 1: Table S9). Motility parameters were significantly lower in the second video recordings relative to the first ones (time point: all *P* < 0.05; Additional file 1: Table S9), which was in line with earlier observations from a pilot experiment where we observed consistent decreases in sperm motility over time. Previously, the time point covariate was only significant for proportion of motile sperm in the experiment testing motility in own and rival seminal fluid (time point: *P* = 0.02; Additional file 1: Table S7), but this factor became consistently significant in the last experiment because slower acquisition times of the equipment increased data collection from 1-2 min to 3 min. We also found a significant interaction between time point and treatment for proportion of motile sperm, VSL and LIN (all *P* < 0.05, Additional file 1: Table S9), suggesting that queen secretions in spermatheca and bursa copulatrix organs preserved sperm motility better over time than Hayes saline.

## Discussion

Our results indicate that the seminal fluid of *A. echinatior* males contains compounds that induce sperm activation, enhance sperm motility, and improve directionality of sperm movement. We show that these effects are similar to those induced by the female reproductive tract fluid [present study and 31] and suggest that opposing gradients of male and female stimulation are likely to be maintained in the provisional bursa copulatrix sperm storage organ. The aim of our study was neither to replicate the natural environment that sperm experience after insemination nor to obtain absolute quantifications of sperm motility. Rather, we quantified the effects of female bursa copulatrix and spermatheca fluids and male seminal fluid while eliminating possible interference or adjustment by other factors that may affect sperm motility in natural female reproductive tracts (e.g. pH, temperature or ion gradients). To our knowledge, our study is the first to experimentally quantify the sex-specific factors that modify sperm competition and sperm storage in a social insect where extreme fertility demands and lack of female re-mating later in life imply that sperm competition dynamics are easier to capture than in mating systems with continuous female promiscuity.

Our results are consistent with differential regulation of sperm motility via own and rival male seminal fluid. We hypothesize that the effects that we observed are adaptive because they allow sperm to increase motility when exposed to rival seminal fluid within the queen genital tract. This response seems straightforward to interpret because seminal fluid of other males is known to incapacitate sperm in *A. echinatior* [21], so reducing the time spent in contact with non-own seminal fluid and pursuing more rapid storage in the spermatheca must have been selected for. In a number of vertebrates and invertebrates, sperm respond to subtle changes in their environment [e.g. pH, temperature or ion gradients and specific female-derived chemoattractants; reviewed in 1, 40, 41], but sperm responses to male-secreted compounds regulating competition between ejaculates were only known in a few vertebrate species [22, 23, 25]. Our findings indicate that *A. echinatior* sperm motility parameters are enhanced by own seminal fluid but much more by non-own seminal fluid and spermathecal fluid, which both derive from non-self somatic tissue from the perspective of focal sperm. This matches a previous study showing that seminal fluid affects sperm viability in a similarly differentiated manner, with own seminal fluid maintaining sperm viability better than rival seminal fluid [21]. This suggests that the molecular mechanisms regulating sperm viability and motility in *A. echinatior* are somehow linked.

Our finding that motility enhancement is relatively modest as long as sperm are only in contact with own seminal fluid is intriguing. Motility is energetically expensive to sustain and likely requires aerobic metabolism, which will lead to the accumulation of reactive oxygen species (ROS) that are damaging for cell viability [42–44]. This may imply a trade-off between increased sperm motility sustained by aerobic ATP production to remain competitive in the race for storage, and sperm viability that is essential for long-term survival in storage and subsequent egg fertilization [45–49]. Recent work in other social insects with long-term sperm storage by queens has indicated that ROS production by sperm represents a significant selective pressure that shaped adaptations to preserve sperm viability. In the honeybee, antioxidative enzymes are found in both male and female reproductive secretions [50, 51] and in both honeybees and *Crematogaster* ants the production of these enzymes in the queen spermatheca is strongly upregulated during the sperm storage process [52–54]. Our study therefore suggests that ROS production may have imposed selection for optimizing rather than maximizing energetic expenditure for individual sperm cells.

Sperm of honeybee drones are known to use both aerobic and anaerobic metabolic pathways upon ejaculation, but primarily anaereobic metabolism during long-term storage in the spermatheca. In this organ oxygen concentrations are very low compared to other queen tissues [48], and similar anoxic conditions occur in queen spermathecae of *Atta* leaf-cutting ants (B. Baer, unpublished observations). In the cricket *Gryllus bimaculatus* sperm are also known to reduce metabolic rates and ROS production by ca. 40% after female storage relative to freshly ejaculated sperm [55]. This may imply that sperm of insects with prolonged sperm storage may have the general capacity to alternate between a slow metabolic state producing ATP via the final steps of glycolysis to maintain viability while avoiding ROS production, and a more active state that incurs costs of ROS production while sustaining the greater energy demands of active sperm competition.

More work will be needed to establish whether similar alternative respiration pathways are operational in *A. echinatior*, and whether increased sperm motility is associated with higher ROS production also in leaf-cutting ants. Recent proteomic work suggests that *A. echinatior* seminal fluid contains a diverse suite of glycolytic and antioxidative enzymes, which are more abundant in the polyandrous *Acromyrmex* lineage than in the seminal fluid of a monandrous *Trachymyrmex* sister lineage (J. Liberti, unpublished PhD thesis), consistent with a greater need for both energy production and ROS control during sperm competition. If further work would confirm that motility induction by rival seminal fluid leads to increased oxidative stress, ROS damage may offer a proximate explanation for the sperm mortality induced by seminal fluid of rival males that was previously identified for both polyandrous attine ants and bees [21]. This could imply that seminal fluid may have been selected to induce metabolic exhaustion of sperm that are not genetically identical, which would seem relatively straightforward because ejaculates of haploid hymenopteran males are clonal, or that sperm use molecular cues from rival seminal fluid to pursue more storage space in spite of metabolic costs.

It is important to remember that the basic characteristics of social hymenopteran mating systems are highly peculiar because there is no connection between competitive processes that affect sperm storage and preferential sperm use for fertilization. This is because sperm are thoroughly mixed once the spermatheca has been filled and no new ejaculates will ever be added. Sperm use for fertilization, often after many years of storage, is therefore a fair raffle as has been explicitly documented for both *Atta* and *Acromyrmex* leaf-cutting ants [56, 57]. This implies that associations between mating order and sperm storage (first- or last male precedence) will affect the overall distribution of potential paternity during sperm storage, but that queens will not be able to differentially use sperm of specific males for fertilization of eggs later on. Also the fact that sibling workers take care of all larval provisioning (thus determining which larvae will develop as sterile workers or fertile future queens) should normally preclude the order of insemination to have more than a mere statistical effect on the general likelihood of paternity. These and other peculiarities of social hymenopteran mating systems have been extensively reviewed elsewhere [27, 28, 58].

When sperm behavior is affected by a trade-off between active respiration and ROS damage, polyandrous queens might encourage sperm motility via spermathecal secretions that reach the bursa copulatrix via diffusion through the spermathecal duct, which would then likely result in the most viable sperm being stored. This hypothetical scenario would be consistent with our finding that female effects on sperm motility are more strongly induced by fluids from the spermatheca than by fluids from the bursa copulatrix, and would also match the production of chemokinetic molecules being associated with the spermathecal glands throughout the Hymenoptera [59–62]. An alternative explanation could be that the pre-storage bursa copulatrix has fewer secretory cells to produce sperm-activating compounds than the spermatheca, which would create a similar chemical gradient of sperm motility-activation from the pre-storage organ to the final spermathecal storage organ. The regulation of sperm metabolism would then be expected to depend on the interaction between molecules present in glandular secretions and the overall oxygen levels in the different compartments of the queen reproductive tract. Diffusion of spermathecal secretions into the bursa copulatrix (where oxygen levels are likely higher) would then particularly increase sperm motility near the spermathecal duct in the distal part of the bursa copulatrix, but once sperm have entered the spermatheca, they would experience low oxygen conditions and slow down metabolism to avoid ROS-induced damage, as recently documented for honeybees [48].

The evolutionary dynamics of sperm competition that we documented and inferred may be comparable to those found in other organisms. In the externally fertilizing sea urchin *Lytechinus variegatus* faster swimming sperm are shorter lived even though they are likely to fertilize more eggs [46], and in the Atlantic salmon (*Salmo salar*) sperm velocity is the primary determinant of fertilization success while sperm longevity is negatively correlated with the probability of fertilization, even though a direct trade-off between velocity and longevity could not be established [63]. In the internally fertilizing fish *Xiphophorus nigrensis* males with faster swimming sperm sire fewer offspring when females store sperm for prolonged periods of time, suggesting that higher motility depletes sperm resources that could also be used for maintenance in storage or ROS-damage repair [45]. Similarly, sperm velocity is negatively correlated with clutch size (a proxy for the duration of sperm storage) across the passerine birds, suggesting once more that sperm motility may trade-off with prolonged sperm survival in storage organs [16].

## Conclusions

We show that the unusual mating system characteristics of social hymenopteran lineages (ants, bees and wasps) that convergently evolved polyandry from monandrous ancestors can select for plasticity in sperm behavior with prompt but differential response to the presence or absence of competing sperm. This regulation may allow spermatozoa to optimize energetic investments in sperm motility, which is likely necessary to achieve sperm storage in competition with sperm from other ejaculates. However, higher motility may well be detrimental for long-term sperm viability after sperm have been stored, so that queen genital tract secretions that encourage sperm competition may be constrained by a sperm trade-off between maximizing both the likelihood of storage and the probability of still being viable after years of storage. Such a trade-off has been documented in solitary animals where female promiscuity is the norm, but seems remarkable in evolutionarily derived social insects that evolved polyandry from strictly monogamous ancestors [64].

## Additional files

Additional file 1: **Tables S1-S9.** Full legends are contained within the file. (DOCX 69 kb)

Additional file 2: Datasets supporting this article. (XLSX 68 kb)

## Abbreviations

AG: accessory glands
AT: accessory testes
ATP: adenosine triphosphate
CASA: computer-assisted sperm analyser
fps: frames per second
LIN: sperm linearity
PC1: first principal component
PCA: principal component analysis
PCR: polymerase chain reaction
ROS: reactive oxygen species
*VAP*: velocity on the average path
VCL: curvilinear velocity
VSL: straight-line velocity
