# Supplementary material for: Rival seminal fluid induces enhanced sperm motility in a polyandrous ant
Source: BMC Evol Biol. 2018 Mar 23;18:28. doi: 10.1186/s12862-018-1144-y (PMC5865361; doi:10.1186/s12862-018-1144-y)
Supplement: Supplementary file 1 — Tables S1-S9. Full legends are contained within the file. (DOCX 69 kb) [file 12862_2018_1144_MOESM1_ESM.docx]

**Electronic Supplementary Materials**

**Rival seminal fluid induces enhanced sperm motility in a polyandrous ant**

Joanito Liberti^1^, Boris Baer^2^ and Jacobus J. Boomsma^1^

**Affiliations:**

^1^Centre for Social Evolution, Department of Biology, University of Copenhagen, Universitetsparken 15, DK-2100, Copenhagen, Denmark

^2^Centre for Integrative Bee Research (CIBER), Department of Entomology, University of California Riverside, CA 92521, United States of America

**Corresponding authors:**

Joanito Liberti ([joanito.liberti@bio.ku.dk](mailto:Joanito.liberti@bio.ku.dk)); Jacobus J. Boomsma ([jjboomsma@bio.ku.dk](mailto:jjboomsma@bio.ku.dk))

**Table S1** Collection data for the *Acromyrmex echinatior* (Ae) colonies used across the experiments. Colony numbers given upon collection in Gamboa, Panama, are given plus the years at which colonies were excavated. The experiments reported were done in 2016, after colonies had been maintained for 2-14 years in Copenhagen rearing rooms.

| **Donor colony** | **Year of collection** |
| --- | --- |
| Ae168 | 2002 |
| Ae226B | 2003 |
| Ae322 | 2006 |
| Ae332 | 2007 |
| Ae342 | 2007 |
| Ae345 | 2007 |
| Ae361 | 2008 |
| Ae372 | 2008 |
| Ae376 | 2008 |
| Ae420A | 2009 |
| Ae506 | 2011 |
| Ae507 | 2011 |
| Ae704 | 2014 |

**Table S2** Combinations of colonies used as male donors in the experiment testing the effects of ejaculate admixture on sperm motility. The entire series of ten trials was done twice using identical colony combinations.

| **Trial** | **Colony of individual male 1** | **Colony of individual male 2** |
| --- | --- | --- |
| 1 | Ae332 | Ae168 |
| 2 | Ae168 | Ae420A |
| 3 | Ae420A | Ae345 |
| 4 | Ae345 | Ae342 |
| 5 | Ae342 | Ae361 |
| 6 | Ae361 | Ae376 |
| 7 | Ae376 | Ae226B |
| 8 | Ae226B | Ae322 |
| 9 | Ae322 | Ae506 |
| 10 | Ae506 | Ae372 |

**Table S3** Combinations of colonies used as male donors in the experiment testing the effects of own and rival seminal fluid on sperm motility. The entire series of ten trials was done twice using identical colony combinations.

| **Trial** | **Focal male donor colony** | **Rival male donor colony** |
| --- | --- | --- |
| 1 | Ae322 | Ae226B |
| 2 | Ae226B | Ae372 |
| 3 | Ae372 | Ae342 |
| 4 | Ae342 | Ae506 |
| 5 | Ae506 | Ae361 |
| 6 | Ae361 | Ae507 |
| 7 | Ae507 | Ae704 |
| 8 | Ae704 | Ae420A |
| 9 | Ae420A | Ae168 |
| 10 | Ae168 | Ae376 |

**Table S4** Combinations of colonies used as male and female donors in the experiment comparing the effects of seminal fluid and queen reproductive tract fluid on sperm motility. The entire series of ten trials was done twice using identical colony combinations.

| **Trial** | **Focal male donor colony** | **Virgin queen donor colony** | **Rival male donor colony** |
| --- | --- | --- | --- |
| 1 | Ae226B | Ae376 | Ae168 |
| 2 | Ae376 | Ae168 | Ae420A |
| 3 | Ae168 | Ae420A | Ae322 |
| 4 | Ae420A | Ae322 | Ae372 |
| 5 | Ae322 | Ae372 | Ae332 |
| 6 | Ae372 | Ae332 | Ae704 |
| 7 | Ae332 | Ae704 | Ae342 |
| 8 | Ae704 | Ae342 | Ae507 |
| 9 | Ae342 | Ae507 | Ae506 |
| 10 | Ae507 | Ae506 | Ae376 |

**Table S5** Combinations of colonies used as male and female donors in the experiment comparing the effects of spermathecal fluid and bursa copulatrix fluid of the same virgin queens on sperm motility. The entire series of ten trials was done twice using identical colony combinations.

| **Trial** | **Focal male donor colony** | **Virgin queen donor colony** |
| --- | --- | --- |
| 1 | Ae322 | Ae420A |
| 2 | Ae420A | Ae226B |
| 3 | Ae226B | Ae332 |
| 4 | Ae332 | Ae704 |
| 5 | Ae704 | Ae342 |
| 6 | Ae342 | Ae506 |
| 7 | Ae506 | Ae372 |
| 8 | Ae372 | Ae376 |
| 9 | Ae376 | Ae168 |
| 10 | Ae168 | Ae322 |

**Table S6** Results of linear mixed-effects models fitted by restricted maximum likelihood in experimental tests of the effect of ejaculate admixture on sperm motility, including results for the three velocity variables (VCL, VAP and VSL) that loaded PC1, and the percentage of variance explained by random effects. DF = degrees of freedom; DF_Den_ = denominator degrees of freedom. Significant results are presented in bold.

| **Dependent variable** | **Fixed effects** | **DF** | **DF_Den_** | **F Ratio** | **p-value** |
| --- | --- | --- | --- | --- | --- |
| Proportion of motile sperm | Treatment | 1 | 106 | 22.49 | **<.0001** |
|  | Time Point | 1 | 106 | 1.62 | 0.20 |
|  | Treatment*Time Point | 1 | 106 | 0.01 | 0.98 |
|  |  |  |  |  |  |
|  | **Random effects** | Experiment replicate | Trial | Residual |  |
|  |  | 0.66% | 4.84% | 94.50% |  |
| PC1 | Treatment | 1 | 106 | 13.69 | **0.0003** |
|  | Time Point | 1 | 106 | 0.03 | 0.85 |
|  | Treatment*Time Point | 1 | 106 | 0.83 | 0.36 |
|  |  |  |  |  |  |
|  | **Random effects** | Experiment replicate | Trial | Residual |  |
|  |  | 0.00% | 5.92% | 94.08% |  |
| VCL | Treatment | 1 | 106 | 10.90 | **0.0013** |
|  | Time Point | 1 | 106 | 0.13 | 0.71 |
|  | Treatment*Time Point | 1 | 106 | 1.35 | 0.25 |
|  |  |  |  |  |  |
|  | **Random effects** | Experiment replicate | Trial | Residual |  |
|  |  | 0.00% | 3.71% | 96.29% |  |
| VAP | Treatment | 1 | 106 | 13.20 | **0.0004** |
|  | Time Point | 1 | 106 | 0.29 | 0.59 |
|  | Treatment*Time Point | 1 | 106 | 0.61 | 0.44 |
|  |  |  |  |  |  |
|  | **Random effects** | Experiment replicate | Trial | Residual |  |
|  |  | 0.00% | 6.92% | 93.08% |  |
| VSL | Treatment | 1 | 106 | 14.67 | **0.0002** |
|  | Time Point | 1 | 106 | 0.13 | 0.72 |
|  | Treatment*Time Point | 1 | 106 | 0.52 | 0.47 |
|  |  |  |  |  |  |
|  | **Random effects** | Experiment replicate | Trial | Residual |  |
|  |  | 0.00% | 6.56% | 93.44% |  |
| LIN | Treatment | 1 | 106 | 5.54 | **0.0204** |
|  | Time Point | 1 | 106 | 0.51 | 0.48 |
|  | Treatment*Time Point | 1 | 106 | 0.46 | 0.50 |
|  |  |  |  |  |  |
|  | **Random effects** | Experiment replicate | Trial | Residual |  |
|  |  | 0.00% | 0.00% | 100.00% |  |

**Table S7** Results of linear mixed-effects models fitted by restricted maximum likelihood in experimental tests of the effects of own and rival male seminal fluid on sperm motility, including results for the three velocity variables (VCL, VAP and VSL) that loaded PC1, and the percentage of variance explained by random effects. DF = degrees of freedom; DF_Den_ = denominator degrees of freedom. Significant results are presented in bold.

| **Dependent variable** | **Fixed effects** | **DF** | **DF_Den_** | **F Ratio** | **p-value** |
| --- | --- | --- | --- | --- | --- |
| Proportion of motile sperm | Treatment | 3 | 142 | 29.74 | **<.0001** |
|  | Time Point | 1 | 142 | 5.92 | **0.02** |
|  | Treatment*Time Point | 3 | 142 | 1.94 | 0.12 |
|  |  |  |  |  |  |
|  | **Random effects** | Experiment replicate | Trial | Residual |  |
|  |  | 0.00% | 10.54% | 89.46% |  |
| PC1 | Treatment | 3 | 142 | 20.47 | **<.0001** |
|  | Time Point | 1 | 142 | 0.02 | 0.89 |
|  | Treatment*Time Point | 3 | 142 | 1.10 | 0.35 |
|  |  |  |  |  |  |
|  | **Random effects** | Experiment replicate | Trial | Residual |  |
|  |  | 11.20% | 4.52% | 84.28% |  |
| VCL | Treatment | 3 | 142 | 16.98 | **<.0001** |
|  | Time Point | 1 | 142 | 0.01 | 0.90 |
|  | Treatment*Time Point | 3 | 142 | 1.01 | 0.39 |
|  |  |  |  |  |  |
|  | **Random effects** | Experiment replicate | Trial | Residual |  |
|  |  | 9.43% | 2.94% | 87.63% |  |
| VAP | Treatment | 3 | 142 | 19.06 | **<.0001** |
|  | Time Point | 1 | 142 | 0.06 | 0.81 |
|  | Treatment*Time Point | 3 | 142 | 1.11 | 0.34 |
|  |  |  |  |  |  |
|  | **Random effects** | Experiment replicate | Trial | Residual |  |
|  |  | 11.42% | 5.05% | 83.53% |  |
| VSL | Treatment | 3 | 142 | 23.56 | **<.0001** |
|  | Time Point | 1 | 142 | 0.08 | 0.77 |
|  | Treatment*Time Point | 3 | 142 | 1.12 | 0.34 |
|  |  |  |  |  |  |
|  | **Random effects** | Experiment replicate | Trial | Residual |  |
|  |  | 11.22% | 6.35% | 82.43% |  |
| LIN | Treatment | 3 | 142 | 8.59 | **<.0001** |
|  | Time Point | 1 | 142 | 0.03 | 0.8536 |
|  | Treatment*Time Point | 3 | 142 | 0.08 | 0.9712 |
|  |  |  |  |  |  |
|  | **Random effects** | Experiment replicate | Trial | Residual |  |
|  |  | 0.00% | 6.55% | 93.45% |  |

**Table S8** Results of linear mixed-effects models fitted by restricted maximum likelihood in experimental tests of the effects of seminal fluid and queen reproductive tract fluid on sperm motility, including results for the three velocity variables (VCL, VAP and VSL) that loaded PC1, and the percentage of variance explained by random effects. DF = degrees of freedom; DF_Den_ = denominator degrees of freedom. Significant results are presented in bold.

| **Dependent variable** | **Fixed effects** | **DF** | **DF_Den_** | **F Ratio** | **p-value** |
| --- | --- | --- | --- | --- | --- |
| Proportion of motile sperm | Treatment | 3 | 142 | 24.18 | **<.0001** |
|  | Time Point | 1 | 142 | 0.39 | 0.53 |
|  | Treatment*Time Point | 3 | 142 | 0.45 | 0.72 |
|  |  |  |  |  |  |
|  | **Random effects** | Experiment replicate | Trial | Residual |  |
|  |  | 0.00% | 17.83% | 82.17% |  |
| PC1 | Treatment | 3 | 142 | 10.72 | **<.0001** |
|  | Time Point | 1 | 142 | 0.22 | 0.64 |
|  | Treatment*Time Point | 3 | 142 | 0.64 | 0.59 |
|  |  |  |  |  |  |
|  | **Random effects** | Experiment replicate | Trial | Residual |  |
|  |  | 0.00% | 21.41% | 78.59% |  |
| VCL | Treatment | 3 | 142 | 5.27 | **0.0018** |
|  | Time Point | 1 | 142 | 0.02 | 0.89 |
|  | Treatment*Time Point | 3 | 142 | 0.95 | 0.42 |
|  |  |  |  |  |  |
|  | **Random effects** | Experiment replicate | Trial | Residual |  |
|  |  | 0.00% | 7.41% | 92.59% |  |
| VAP | Treatment | 3 | 142 | 11.53 | **<.0001** |
|  | Time Point | 1 | 142 | 0.34 | 0.56 |
|  | Treatment*Time Point | 3 | 142 | 0.42 | 0.74 |
|  |  |  |  |  |  |
|  | **Random effects** | Experiment replicate | Trial | Residual |  |
|  |  | 0.00% | 22.33% | 77.67% |  |
| VSL | Treatment | 3 | 142 | 12.04 | **<.0001** |
|  | Time Point | 1 | 142 | 0.80 | 0.37 |
|  | Treatment*Time Point | 3 | 142 | 0.38 | 0.77 |
|  |  |  |  |  |  |
|  | **Random effects** | Experiment replicate | Trial | Residual |  |
|  |  | 0.22% | 28.07% | 71.71% |  |
| LIN | Treatment | 3 | 142 | 2.90 | **0.0373** |
|  | Time Point | 1 | 142 | 2.44 | 0.12 |
|  | Treatment*Time Point | 3 | 142 | 1.11 | 0.35 |
|  |  |  |  |  |  |
|  | **Random effects** | Experiment replicate | Trial | Residual |  |
|  |  | 0.99% | 7.67% | 91.34% |  |

**Table S9** Results of linear mixed-effects models fitted by restricted maximum likelihood in experimental tests of the effects of the fluids contained within the spermatheca and the bursa copulatrix of virgin queens on sperm motility, including results for the three velocity variables (VCL, VAP and VSL) that loaded PC1, and the percentage of variance explained by random effects. DF = degrees of freedom; DF_Den_ = denominator degrees of freedom. Significant results are presented in bold.

| **Dependent variable** | **Fixed effects** | **DF** | **DF_Den_** | **F Ratio** | **p-value** |
| --- | --- | --- | --- | --- | --- |
| Proportion of motile sperm | Treatment | 2 | 105 | 41.54 | **<.0001** |
|  | Time Point | 1 | 105 | 11.57 | **0.0009** |
|  | Treatment*Time Point | 2 | 105 | 4.91 | **0.0092** |
|  |  |  |  |  |  |
|  | **Random effects** | Experiment replicate | Trial | Residual |  |
|  |  | 0.00% | 10.65% | 89.35% |  |
| PC1 | Treatment | 2 | 105 | 28.28 | **<.0001** |
|  | Time Point | 1 | 105 | 6.98 | **0.0095** |
|  | Treatment*Time Point | 2 | 105 | 3.03 | 0.05 |
|  |  |  |  |  |  |
|  | **Random effects** | Experiment replicate | Trial | Residual |  |
|  |  | 0.00% | 32.76% | 67.24% |  |
| VCL | Treatment | 2 | 105 | 25.96 | **<.0001** |
|  | Time Point | 1 | 105 | 4.46 | **0.0371** |
|  | Treatment*Time Point | 2 | 105 | 1.88 | 0.16 |
|  |  |  |  |  |  |
|  | **Random effects** | Experiment replicate | Trial | Residual |  |
|  |  | 0.00% | 27.54% | 72.46% |  |
| VAP | Treatment | 2 | 105 | 25.00 | **<.0001** |
|  | Time Point | 1 | 105 | 7.57 | **0.007** |
|  | Treatment*Time Point | 2 | 105 | 3.01 | 0.05 |
|  |  |  |  |  |  |
|  | **Random effects** | Experiment replicate | Trial | Residual |  |
|  |  | 0.00% | 31.96% | 68.04% |  |
| VSL | Treatment | 2 | 105 | 29.49 | **<.0001** |
|  | Time Point | 1 | 105 | 8.06 | **0.0054** |
|  | Treatment*Time Point | 2 | 105 | 3.96 | **0.0219** |
|  |  |  |  |  |  |
|  | **Random effects** | Experiment replicate | Trial | Residual |  |
|  |  | 0.00% | 36.51% | 63.49% |  |
| LIN | Treatment | 2 | 105 | 11.55 | **<.0001** |
|  | Time Point | 1 | 105 | 5.73 | **0.0184** |
|  | Treatment*Time Point | 2 | 105 | 7.08 | **0.0013** |
|  |  |  |  |  |  |
|  | **Random effects** | Experiment replicate | Trial | Residual |  |
|  |  | 0.00% | 7.43% | 92.57% |  |
